# Detection and quantification of STLV-1 and SFV proviral load in blood and saliva of naturally infected non-human primates

**DOI:** 10.1186/1742-4690-12-S1-P4

**Published:** 2015-08-28

**Authors:** Sandrine Alais, Amandine Pasquier, Jocelyn Turpin, Rejane Rua, Antoine Gessain, Romain Lacoste, Renaud Mahieux

**Affiliations:** 1 Equipe Oncogenèse Rétrovirale, International Center for Research in Infectiology, INSERM U1111 – CNRS UMR5308, France; 2 Equipe labellisée “Ligue Nationale Contre le Cancer”, International Center for Research in Infectiology, INSERM U1111 – CNRS UMR5308, France; 3 International Center for Research in Infectiology, INSERM U1111 – CNRS UMR5308, France; 4 Ecole Normale Supérieure de Lyon, Lyon, France; 5 Université Lyon 1, Lyon, 69364 Cedex 07, France; 6 Ecole Pratique des Hautes Etudes, Pasteur Institute, Paris, 75724 Cedex 15, France; 7 Epidémiologie et Physiopathologie des Virus Oncogènes, CNRS UMR 3569, Pasteur Institute, Paris, 75724 Cedex 15, France; 8 Station de Primatologie-UPS846-CNRS, Rousset sur Arc, 13790, France

## 

Simian T Lymphotropic Virus type 1 (STLV-1) and Simian Foamy Virus (SFV) retroviruses infect Old World non-human primates (NHP) and humans. Inter-human transmission has been described for HTLV-1 but not for SFV. SFV infection is asymptomatic in its hosts, while STLV-1 and its human counterpart HTLV-1 are the etiologic agents of Adult T-cell Leukemia/Lymphoma. Both STLV-1 and SFV can be zoonotically transmitted from NHP to humans through severe bites, thus involving contact between virus-containing saliva in the donor and blood in the recipient. Surprisingly, while the presence of both SFV RNA and DNA has been characterized into the saliva of NHP, neither STLV-1 DNA, nor STLV-1 RNA was quantified. Thus, the goal of our study was to search for STLV-1 provirus in the cells present in the saliva of NHP and then to quantify the proviral load of both viruses. We took advantages of a cohort of 45 papio anubis, naturally infected by STLV-1. We first assessed SFV infection and then potential SFV/STLV-1 co-infections. To this end, we designed semi-nested PCR and qPCR protocols (1) to diagnose infection and (2) to quantify STLV-1 and/or SFV proviral load in peripheral blood cells and in saliva. First, STLV-1 provirus was detected by semi-nested PCR in 8/10 blood samples tested, but only in the saliva of 1/10 NHP who had a high STLV-1 proviral load in peripheral blood cells. SFV DNA was detected by nested-PCR in blood samples from 10/10 baboons and in the saliva of 8/10 animals. A second study performed with 20 animals will be presented. We will show whether a correlation exists between blood and saliva STLV-1/SFV proviral load and whether infection with one retrovirus impacts proviral load of the other. Altogether, our current results suggest that SFV is more frequently present in saliva than STLV-1. This should impact the ability of both viruses to be zoonotically transmitted through bites.

